# Radiobiological Characterization of ^64^CuCl_2_ as a Simple Tool for Prostate Cancer Theranostics

**DOI:** 10.3390/molecules23112944

**Published:** 2018-11-11

**Authors:** Joana Fernandes Guerreiro, Vítor Alves, Antero José Abrunhosa, António Paulo, Octávia Monteiro Gil, Filipa Mendes

**Affiliations:** 1 Centro de Ciências e Tecnologias Nucleares, Instituto Superior Técnico, Universidade de Lisboa, Estrada Nacional 10 (km 139,7), 2695-066 Bobadela LRS, Portugal; apaulo@ctn.tecnico.ulisboa.pt (A.P.); ogil@ctn.tecnico.ulisboa.pt (O.M.G.); fmendes@ctn.tecnico.ulisboa.pt (F.M.); 2 Instituto de Ciências Nucleares Aplicadas à Saúde, Universidade de Coimbra, Pólo de Ciências da Saúde, Az. Sta. Comba, 3000-548 Coimbra, Portugal; vhpalves@gmail.com (V.A.); antero@pet.uc.pt (A.J.A.)

**Keywords:** copper-64 chloride, theranostics, prostate cancer, radiobiology, auger therapy

## Abstract

^64^CuCl_2_ has recently been proposed as a promising agent for prostate cancer (PCa) theranostics, based on preclinical studies in cellular and animal models, and on the increasing number of human studies documenting its use for PCa diagnosis. Nevertheless, the use of ^64^CuCl_2_ raises important radiobiological questions that have yet to be addressed. In this work, using a panel of PCa cell lines in comparison with a non-tumoral prostate cell line, we combined cytogenetic approaches with radiocytotoxicity assays to obtain significant insights into the cellular consequences of exposure to ^64^CuCl_2_. PCa cells were found to exhibit increased ^64^CuCl_2_ uptake, which could not be attributed to increased expression of the main copper cellular importer, hCtr1, as had been previously suggested. Early DNA damage and genomic instability were also higher in PCa cells, with the tumoral cell lines exhibiting deficient DNA-damage repair upon exposure to ^64^CuCl_2_. This was corroborated by the observation that ^64^CuCl_2_ was more cytotoxic in PCa cells than in non-tumoral cells. Overall, we showed for the first time that PCa cells had a higher sensitivity to ^64^CuCl_2_ than healthy cells, supporting the idea that this compound deserved to be further evaluated as a theranostic agent in PCa.

## 1. Introduction

Prostate cancer (PCa) is the cancer type with the highest incidence in the male population of the United States and in the Northern and Western countries of the European Union, being the second highest cancer-related cause of death among men in those countries [1,2]. Diagnosed at an early stage, PCa is a manageable disease, but the metastatic castration-resistant stage of the illness is usually considered incurable [3]. Despite being one of the most commonly used positron emission tomography (PET) tracers in oncology, ^18^F-Fludeoxyglucose has limited sensitivity for the detection of primary PCa and recurrent disease [4]. Currently, ^99m^Tc-methylene diphosphonate bone scintigraphy is the standard method used to detect bone metastases, while ^18^F-choline and ^11^C-choline PET offer high sensitivity and specificity to detect lymph node involvement and distant metastases, as well as disease recurrence, being well established tracers for those purposes [4,5]. ^68^Ga-prostate-specific membrane antigen targeted radiopharmaceuticals, which are designed to recognize a membrane glycoprotein that is highly expressed in human PCa cells, have also emerged as having tremendous potential for the detection and re-staging of PCa after biochemical recurrence, offering superior contrast and sensitivity than ^18^F-choline [4]. However, despite the availability of these probes which have proven to be valuable tools for the management of PCa, the imaging role of PET has been limited, particularly in primary PCa and disease recurrence detection, by variable accuracy of the radiotracers in discriminating cancer from normal prostate tissue or benign hyperplasia [4,5,6]. Current therapeutic alternatives to treat castration-resistant PCa include the use of chemotherapy, novel hormone therapies targeting androgen signaling, and the use of the radionuclide radium-223 for bone pain palliation [3]. However, these strategies focus only on the increase of the patients’ lifespan and improvement of the patients’ quality of life, as the disease remains fatal [3]. Therefore, there is still a medical need for more efficient diagnostic methods and novel therapy alternatives that can overcome these limitations, in particular novel radiopharmaceuticals with the potential to be used simultaneously for imaging diagnosis and targeted therapy, that is, with theranostics potential [7].

In the above-mentioned context, several copper isotopes are available for cancer imaging and therapy [8], and among these, the cyclotron-produced ^64^Cu is considered one of the most versatile radionuclides that has theranostic potential [8,9]. The positron emission (β^+^) of ^64^Cu allows high resolution PET imaging for diagnosis and follow-up of antitumoral therapy, with a relatively low dose burden to the patients that reduces dosimetry concerns [8,9]. Higher doses can also be used for radionuclide therapy, through the combined emission of beta minus particles (β^−^) and Auger electrons [8,9]. In addition, its relatively long half-life of 12.7 h provides a suitable timeframe to allow for both imaging and therapeutic applications [8]. Copper plays an important role in many biological processes, and copper homeostasis in the cell is a tightly regulated process that is mainly dependent on the major high affinity copper influx transporter in mammalian cells, human copper transporter 1 (hCtr1) [10]. Perturbations of cellular copper homeostasis are known to be associated with various pathological conditions, including cancer [11], where copper has been shown to play a critical role in cell proliferation, angiogenesis, and tumor growth [12]. Considering that the identification of new biomarkers is a major area of ongoing research, copper metabolism emerges as an interesting potential imaging biomarker of cancer, which can be explored using the different copper isotopes currently available.

Despite the biological relevance of copper, only a limited number of ^64^Cu-based compounds have been explored, with the most relevant one, ^64^Cu-diacetyl-bis N4-methylthiosemicarbazone (ATSM), having shown promise in clinical trials as a hypoxia selective tracer and as an indicator of response to treatment and tumor recurrence [13]. Despite this success, its biological effects and the cellular and molecular mechanisms affected by exposure to ^64^Cu remain largely unknown. In fact, only a few pre-clinical studies have been published addressing the radiobiological implications and therapeutic potential of ^64^Cu-ATSM [14,15,16]. Moreover, recent studies have suggested that the simplest form of ^64^Cu, ^64^CuCl_2_, has unique advantages compared with other ^64^Cu-based compounds [8,13]. Preclinical studies in cellular and animal models have demonstrated that ^64^CuCl_2_ has potential as a theranostic agent in several human malignancies, including PCa, glioblastoma, and melanoma [17,18,19,20]. In addition, *hCTR1*-expressing tumor cell lines and xenografts were found to exhibit increased ^64^CuCl_2_ uptake, suggesting that this compound has potential for the treatment of tumors expressing high levels of *hCTR1* [18,21]. Based on the potential of copper metabolism as an imaging biomarker, small-scale human studies have since revealed promising results for staging of PCa and diagnosis of recurrent disease using ^64^CuCl_2_ PET/Computed Tomography (PET/CT), with no adverse pharmacological effects reported in the subjects participating in the studies [22,23].

Overall, while previous findings support further investigation of ^64^CuCl_2_ as a radiopharmaceutical for PCa theranostics, its use also raises radiobiological concerns, intrinsic to its high radiotoxicity, and which have yet to be addressed. In this work, we assessed the effects of exposure to ^64^CuCl_2_ on human prostate cells, using normal and cancer cell lines, in order to obtain significant insights into some of the cellular consequences of exposure to ^64^CuCl_2_, which are important to guide its rational use as a theranostic radiopharmaceutical. Our findings also help to explain the underlying biochemical basis for some of the observations made in pre-clinical and human studies suggesting that ^64^CuCl_2_ has potential as a theranostic agent for PCa.

## 2. Results

### 2.1. ^64^CuCl_2_ Exhibits Increased Uptake in PCa Cell Lines

**Figure 1:**
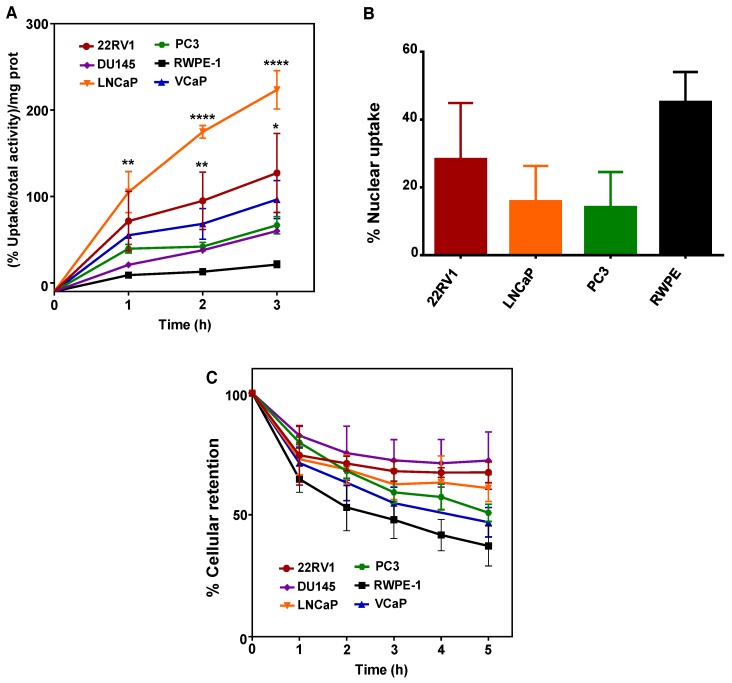
Cellular uptake, nuclear uptake, and cellular retention of ^64^CuCl_2_ in human prostate cell lines. (**A**) The cellular uptake of ^64^CuCl_2_ was determined on a panel of prostate cancer (PCa) (22RV1, DU145, LNCaP, PC3, and VCaP) cell lines and on a non-tumoral (RWPE-1) cell line and is represented as the percentage of cell-associated radioactivity per milligram (mg) of protein over time. (**B**) The nuclear uptake of ^64^CuCl_2_ was determined on selected PCa (22RV1, LNCaP, and PC3) cell lines and on the non-tumoral cell line after 3 h of exposure and is represented as the percentage of cell-associated activity. (**C**) The cellular efflux of ^64^CuCl_2_ in the same panel of prostate cell lines (as in A) is shown as the percentage of cellular retention over a period of 5 h. Statistical significance was calculated using one-way ANOVA, followed by Tukey’s test in comparison with RWPE-1 cells (* *p* ≤ 0.05, ** *p* ≤ 0.01, **** *p* ≤ 0.0001). The results presented were calculated from independent biological replicates (n ≥ 3 for A and n = 2 for B and C) and are given as the mean ± S.E.M.

To explore if ^64^CuCl_2_ would be able to enter into PCa cells as previously suggested by animal studies using human PCa xenografts [18], cellular uptake was assessed on a panel of PCa cell lines derived from bone (22RV1, PC3, and VCaP), brain (DU145) or lymph node (LNCaP) metastasis, using an immortalized, non-tumoral prostate cell line as a control (RWPE-1). ^64^CuCl_2_ uptake was expressed as the percentage of cell-associated radioactivity normalized to the amount of protein, to account for differences in cellular growth between the cell lines. The results obtained showed that cellular uptake increased as a function of incubation time for all tumoral cell lines, but not for the non-tumoral line (Figure 1A). After 3 h of incubation, LNCaP cells exhibited the highest uptake, while the 22RV1 cell line also displayed a significant increase in ^64^CuCl_2_ uptake in comparison with RWPE-1 cells. Even though there was a clear increase in ^64^CuCl_2_ uptake in the VCaP, DU145, and PC3 cell lines in relation to the non-tumoral cell line, particularly at 3 h of incubation, this was found not to be statistically significant.

Since the therapeutic efficiency of Auger emitters has been proposed to be dependent on the emitters ability to reach the nucleus [24,25], the nuclear uptake of ^64^CuCl_2_ was also evaluated after 3 h of incubation with ^64^CuCl_2_ (Figure 1B). Surprisingly, the non-tumoral cell line showed the highest percentage of nuclear uptake, even though significant ^64^CuCl_2_ nuclear uptake was also observed in PCa cells. However, if we took into account the overall cellular uptake of the compound determined for the different cell lines (Figure 1A), the highest percentage of nuclear uptake observed in RWPE-1 cells did not correspond to a higher amount of ^64^CuCl_2_ reaching the nucleus. In reality, both 22RV1 and LNCaP cells were expected to have more nuclear ^64^CuCl_2_ (with values of the percentage of nucleus-associated radioactivity normalized to the amount of protein in the range of 35.9 and 35.3 for 22RV1 and LNCaP cells, respectively) than the PC3 and RWPE-1 cell lines (with estimated values of 9.4 and 9.6, respectively). Finally, to examine whether the tumoral and non-tumoral cell lines might also exhibit different profiles of intracellular retention of ^64^CuCl_2_, we performed efflux experiments. We observed a continuous and moderate washout for all cell lines throughout time that was, albeit not statistically significant, slightly more pronounced for RWPE-1 cells (Figure 1C). Thus, ^64^CuCl_2_ was taken up preferentially by PCa cells, compared with non-tumoral cells, while no significant differences were observed for its cellular retention.

### 2.2. Increased ^64^CuCl_2_ Uptake Does Not Reflect Differential Expression of Copper Transporters

**Figure 2:**
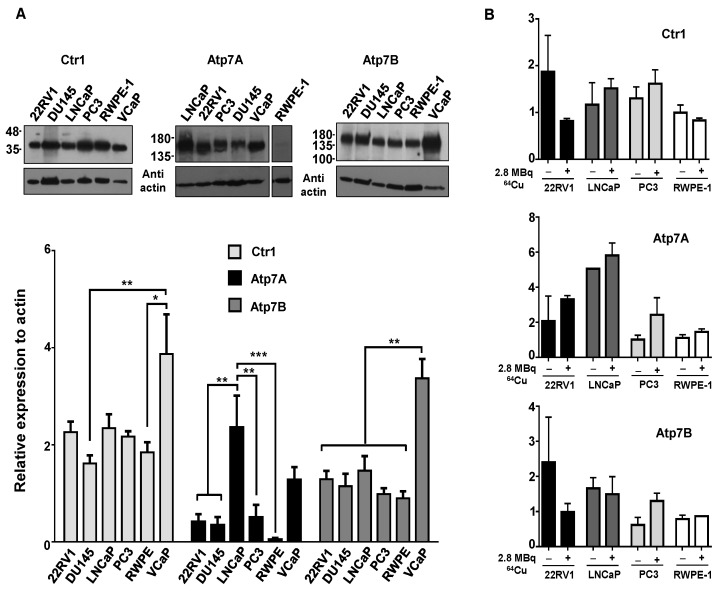
Expression of copper transporters in human prostate cell lines. (**A**) Western blot analysis was used to examine the basal protein expression levels of the copper importer hCtr1, and of the efflux transporters Atp7A and Atp7B in the panel of PCa cell lines under study. Actin was used as a loading control. Statistical significance was calculated using one-way ANOVA, followed by Tukey’s test (* *p* ≤ 0.05, ** *p* ≤ 0.01, *** *p* ≤ 0.001). (**B**) Western blot was performed to determine the protein levels of the same transporters (hCtr1, Atp7A, and Atp7B) after exposure to ^64^CuCl_2_. Cells were grown in 6-well plates and protein lysates were prepared immediately prior (control) or after 3 h of exposure to 2.8 MBq of ^64^CuCl_2_. The results were calculated from independent biological replicates (n ≥ 4 for A and n = 2 for B) and are given as the mean ± S.E.M.

Taking into consideration the preclinical studies that have demonstrated an increased tumoral uptake of ^64^CuCl_2_ in PCa cell lines and tumor xenografts expressing *hCTR1* [18,26], we next investigated whether the increased ^64^CuCl_2_ uptake observed in the tumoral cell lines (Figure 1A) could be correlated with higher levels of copper transporters, using western blot followed by densitometry analysis. In control conditions, the results showed that hCtr1 protein levels did not differ significantly among the different cell lines (Figure 2A, top left panel and lower panel), with the exception of the VCaP cell line which exhibited higher levels of the protein compared with the DU145 and RWPE-1 cell lines. Of note, hCtr1 was detected as two distinct bands with different sizes, one having about 35 kDa (Figure 2A and Figure S1) and the other 70 kDa (Supplementary Figure S1), which, nevertheless, showed similar relative expression levels. In addition, we determined the protein levels of the two major intracellular copper transporters, Atp7A and Atp7B. Atp7A expression was found to be significantly higher in LNCaP cells than in the majority of the other cell lines (Figure 2A, top middle panel and lower panel). For Atp7B, VCaP cells exhibited significantly increased protein levels of this transporter when compared with all the other cell lines (Figure 2A, top right panel and lower panel). After exposure to ^64^CuCl_2_, no significant changes were found on the expression of any of the transporters (Figure 2B). As such, under our conditions, increased uptake of ^64^CuCl_2_ could not be correlated with differential expression of any of the copper transporters analyzed, including hCtr1.

### 2.3. ^64^CuCl_2_ Induces DNA Damage in PCa Cells

**Figure 3:**
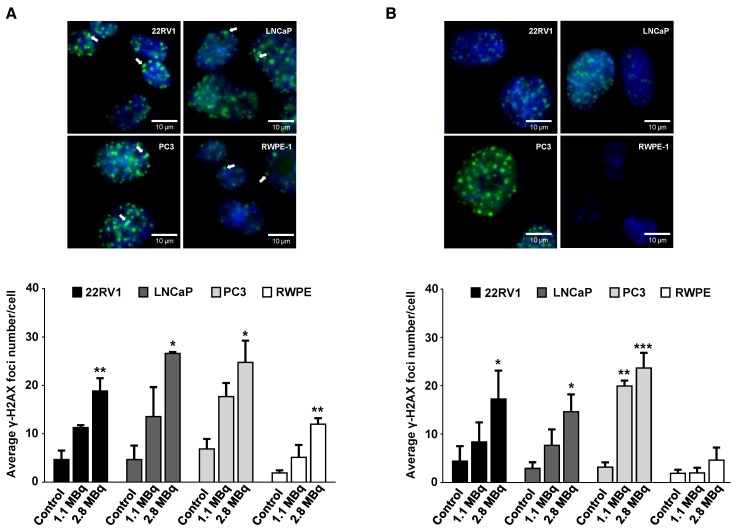
Genotoxic effect of ^64^CuCl_2_ exposure in human prostate cell lines evaluated by the γ-H2AX assay. (**A**) Representative fluorescence microscopy images of γ-H2AX foci in the PCa (22RV1, LNCaP, and PC3) and non-tumoral (RWPE-1) cell lines after 3 h of exposure to 2.8 MBq of ^64^CuCl_2_ (top left panel). Quantification (bottom left panel) of the average number of γ-H2AX foci in untreated cells or cells exposed for 3 h to 1.1 or 2.8 MBq of ^64^CuCl_2_. DAPI staining was used to visualize the nucleus, while immunostaining for γ-H2AX was used for foci detection. Cells were visualized under oil immersion at 64X magnification and arrows indicate the presence of cluster foci. (**B**) Representative fluorescence microscopy images of γ-H2AX foci in the indicated cell lines after 24 h post-exposure to 2.8 MBq of ^64^CuCl_2_ (top right panel). Quantification (bottom right panel) of the average number of γ-H2AX foci in untreated cells or ^64^CuCl_2_ exposed cells after 24 h of recovery. Cells were incubated (or not as a control) in medium containing 1.1 or 2.8 MBq of ^64^CuCl_2_ for 3 h before fresh medium was added and the cells were allowed to recover for 24 h. Statistical significance was calculated using one-way ANOVA, followed by Tukey’s test. Untreated cells were used as a control (* *p* ≤ 0.05, ** *p* ≤ 0.01, *** *p* ≤ 0.001). The results were calculated from independent biological replicates (n = 3) and are given as the mean ± S.E.M.

After initial evaluation of the cellular and nuclear uptake and retention profiles of ^64^CuCl_2_, its genotoxicity was assessed in selected tumoral cell lines and in non-tumoral cells. Considering the results previously obtained (Figure 1A), we selected tumoral cell lines with a high (LNCaP), intermediate (22RV1), and low (PC3) level of ^64^CuCl_2_ uptake to proceed with our studies. The ability of ^64^CuCl_2_ to induce the formation of double-strand breaks (DSBs) in vivo was then assessed using the γ-H2AX assay [27]. Exposure to ^64^CuCl_2_ (2.8 MBq) for 3 h led to a significant increase in the average number of foci for all cell lines (Figure 3A), while a lower dose of 1.1 MBq led to a clear, yet not statistically significant, increase in foci number. Additionally, it was also possible to detect the presence of “streaked” foci, typically observed upon induction of clustered DSBs by high-linear energy transfer (LET) Auger electrons (Figure 3A) [28].

Remarkably, DNA damage induced in the RWPE-1 cell line seemed less pronounced than the damage observed for the tumoral cell lines, even when compared with the PC3 cell line that did not exhibit significantly different cellular or nuclear uptake (Figure 1A,B). DNA damage in terms of DSBs was further assessed after cells had been given 24 h to repair the ^64^CuCl_2_-induced lesions. Notably, after cellular repair, only the non-tumoral cell line was able to reduce the average foci number to the levels of untreated control cells (Figure 3B). 22RV1 and LNCaP cells were largely unable to repair the lesions induced by exposure to ^64^CuCl_2_, but this effect was even more pronounced for the PC3 cell line, where a significant increase in the number of foci was also observed after exposure to a lower dose (1.1 MBq) of ^64^CuCl_2_. This indicates that ^64^CuCl_2_ was able to efficiently induce DNA damage in the tumoral cell lines. Moreover, in those cell lines, in contrast with the non-tumoral cell line, the genomic damage induced exceeded the cellular capacity for its repair.

### 2.4. ^64^CuCl_2_ Induces Genomic Instability in PCa Cells

**Figure 4:**
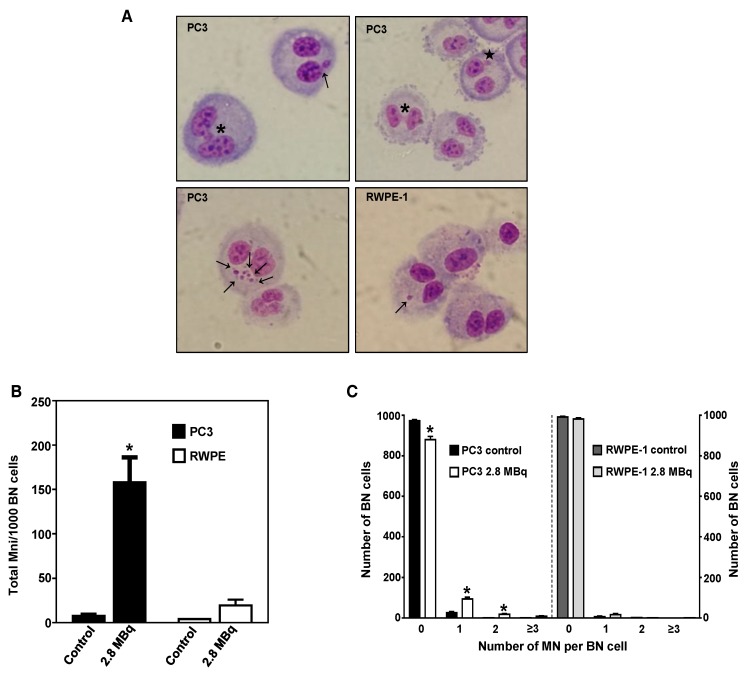
Genotoxic effect of ^64^CuCl_2_ in human prostate cell lines cells evaluated by the cytokinesis-blocked micronucleus (CBMN) assay. (**A**) Representative light microscopy images of complex DNA damage in the tumoral PC3 (top and bottom left panels) and non-tumoral RWPE-1 (bottom right panel) cell lines after 3 h of exposure to 2.8 MBq of ^64^CuCl_2_. The arrows indicate the presence of MNi, the asterisk indicates the presence of a nuclear bridge, and the star indicates the presence of a nuclear bud. Giemsa staining was used to visualize the nuclei and cytoplasm under 40X magnification. (**B**) Quantification of the average number of MNi per 1000 BN PC3 and RWPE-1 control cells or cells exposed to 2.8 MBq of ^64^CuCl_2_. (**C**) Average distribution of MN per 1000 BN PC3 and RWPE-1 cells exposed (or not exposed for control cells) to 2.8 MBq of ^64^CuCl_2_. Statistical significance was calculated using two-tailed Student’s t-test with untreated cells as the control (* *p* ≤ 0.05). The results were calculated from independent biological replicates (n = 2) and are given as the mean ± S.E.M.

The genotoxic effects induced by ^64^CuCl_2_ were further evaluated using the cytokinesis-blocked micronucleus (CBMN) assay, as in Reference [29], in the cell lines exhibiting the highest and lowest radiosensitivity previously determined using the γ-H2AX assay (Figure 4A,B), PC3 and RWPE-1 cell lines. The results revealed a significant increase in the total number of micronuclei (MNi) after exposure to ^64^CuCl_2_ (2.8 MBq) in PC3 cells, when compared to non-exposed cells, which did not occur in RWPE-1 cells (Figure 4A,B). In addition, cells exposed to 2.8 MBq of ^64^CuCl_2_ exhibited increased cellular damage expressed by the occurrence of multiple MNi per BN cell, and the presence of nucleoplasmic bridges and nuclear buds (Figure 4A,C). This indicated that exposure to ^64^CuCl_2_ led to a significant increase in chromosomal damage and genomic instability in PCa cells, but not in immortalized prostate cells.

### 2.5. ^64^CuCl_2_ Exhibits Potent Anti-Proliferative Activity PCa Cells

**Figure 5:**
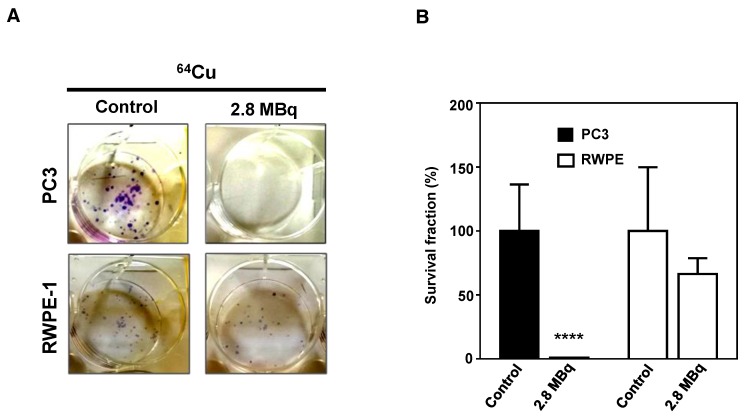
Inhibitory effect of ^64^CuCl_2_ exposure on proliferation in human prostate cell lines evaluated by the clonogenic assay. (**A**) Representative images and (**B**) quantification of the survival fractions of PC3 and RWPE-1 cells after exposure to 2.8 MBq of ^64^CuCl_2_. Statistical significance was calculated using two-tailed Student’s t-test, with untreated cells as the control (**** *p* ≤ 0.0001). The results were calculated from independent biological replicates (n = 3) and are given as the mean ± S.E.M.

Finally, to verify if the genomic damage induced by ^64^CuCl_2_ could elicit a potential antitumoral effect that could be explored for targeted PCa therapy, the radiosensitivity of PC3 and RWPE-1 cells was assessed using the clonogenic assay [30] in cells exposed, or not, to 2.8 MBq of ^64^CuCl_2_ for 3 h. The results showed that there was considerable loss of cellular proliferation ability for PC3 cells exposed to the compound (Figure 5). Contrastingly, RWPE-1 cells showed only a slight impairment in cellular proliferation capacity. These results suggested that the radiotoxic effect of ^64^CuCl_2_ was considerably more pronounced in the tumoral cell line, indicating the potential of this compound to treat prostate malignancies while sparing healthy tissues.

## 3. Discussion

In recent years, a few studies have emerged that aimed to evaluate in human subjects the potential of ^64^CuCl_2_ PET/CT in staging of patients with PCa [23] or in the detection of PCa recurrence [22,31]. The studies were prompted by the promising results obtained in preclinical studies in cellular and animal models, which had suggested that ^64^CuCl_2_ had potential as a theranostic agent in PCa [18,26]. However, at large, none of these previous studies addressed the potential of ^64^CuCl_2_ to be used for PCa targeted therapy, having focused mostly on its use for imaging purposes. Moreover, the cellular effects resulting from exposure to ^64^Cu, which have important clinical implications for both tumoral and healthy tissues, remain largely unknown. Thus, in this work, we set out to evaluate for the first time, the radiobiological effects resulting from exposure to ^64^CuCl_2_ in prostate cells and contribute to the assessment of the potential of this compound to be used for PCa theranostics. The systematic comparison of ^64^CuCl_2_ uptake in a panel of PCa cell lines and in an immortalized epithelial cell line used to study benign hyperplasia [32] revealed that PCa cells exhibited, in general, higher ^64^Cu uptake than non-tumoral cells. This observation provides a cellular framework to the observation that ^64^CuCl_2_ provides images with a very good contrast between malignant lesions and the adjacent non-diseased tissue, which is a limitation of some of the current imaging techniques in use for PCa diagnosis [5,31,33].

We also investigated whether increased ^64^CuCl_2_ uptake could be correlated with expression of the main cellular copper importer, hCtr1, since several studies had previously suggested that increased ^64^CuCl_2_ uptake in different tumors, such as melanoma [17], breast [21] and prostate [18,26] cancers, was dependent on *CTR1* overexpression. However, in our study, no such correlation was found. Albeit surprising at first glance, these results were not entirely unexpected. First, hCtr1 protein levels were previously found to be similar between the DU145, PC3, and RWPE-1 cells lines [26]. Secondly, ^64^CuCl_2_ uptake assessment in animal models harboring human cancer xenografts of several malignancies had shown no correlation between tumor uptake of the radiotracer and *CTR1* expression [34]. Finally, most of the published works investigated the effect of *CTR1* expression on ^64^Cu uptake upon overexpression [21] or downregulation [26] of this importer, and not through analysis of its native expression as done in this study. Therefore, our work provides additional evidence that *CTR1* expression may not be universally increased in cancer [35], and that further investigation is required to establish if *CTR1* overexpression indeed underlies increased uptake of ^64^CuCl_2_ in cancer tissues and if hCtr1 could be used as an appropriate biomarker to predict this compound’s efficacy and monitor therapy in a clinical setting. Given what we established, it remained unclear why PCa cell lines exhibited increased ^64^CuCl_2_ uptake. In cancer, copper has been shown to play a role in cell proliferation and tumor growth [12], and the observation that knockdown of hCtr1 led to growth inhibition of PC3 and DU145-derived tumors in animal models suggested that the importer plays an important role in copper uptake required for rapid proliferation of prostate malignant cells [26]. However, differences in proliferation alone cannot explain the ^64^CuCl_2_ uptake profiles obtained in this work. For instance, the cell line that in our experimental conditions and in agreement with the literature [32], had the fastest growth rate, the PC3 cell line, exhibited one of the lowest uptakes, while the LNCaP cell line, which grew more slowly, had a significantly higher uptake. As such, this is a question which remains worthy of further investigation.

A dosimetric study in humans has recently reported that the dose absorbed by PCa lesions is low, hypothesizing that the therapeutic potential of ^64^CuCl_2_ is largely dependent on the cytotoxic effects of the Auger electrons, rather than on the beta minus particles emitted [31]. Thus, from a therapeutic point of view, in order to be effective, ^64^CuCl_2_ must be able to reach the nucleus, since the cytotoxic ability of Auger emitters has been shown to be dependent on the emitters proximity to the DNA [24,25]. We demonstrated that ^64^CuCl_2_ was indeed able to reach that cellular compartment and induce significant genotoxicity and cytotoxicity in PCa cells, targeting both castration resistance (22RV1 and PC3) and hormone naive (LNCaP) PCa. This radiopharmaceutical was also found to induce less genetic damage in non-tumoral cells, than the one observed in PCa cells. More significantly, PCa cells were shown to exhibit deficient DNA-damage repair capacity as evidenced by persistence of γ-H2AX foci and by a significant increase in the number of MNi after temporary exposure to ^64^CuCl_2_. It was also evident in the γ-H2AX assay that ^64^CuCl_2_ was able to induce the formation of clustered DSBs, comparable to the ones typically induced by high-LET radiation, and which are harder to repair than the isolated DSBs usually induced by low-LET radiation [36]. In contrast, non-tumoral cells were able to efficiently repair the lesions induced by ^64^CuCl_2_ and were noticeably more resistant to ^64^CuCl_2_-induced cytotoxicity than PCa cells. Overall, the lower DNA damage observed in the non-tumoral cell line could be explained by decreased ^64^CuCl_2_ bioavailability resulting from the lower copper uptake exhibited by this cell line in comparison to some of the PCa cell lines (namely, LNCaP and 22RV1). However, considering that all tested PCa cell lines exhibited similar DSBs induction immediately after exposure to ^64^CuCl_2_, even though the cell lines had different ^64^Cu uptake profiles, the same did not seem to hold true to explain the radiosensitivity of the cells.

It is likely that the higher sensitivity of PCa cells, and PC3 cells in particular, to ^64^CuCl_2_ was related to the DNA repair deficiency observed in this study. In fact, the number of DNA lesions formed upon exposure to a radionuclide is a balance between the repaired lesions and the new lesions formed by the radionuclide that remained inside the cells [25,37]. In that context, the behavior exhibited by the different cell lines could be related to the p53 status of the cells, since in prostate cancer, deregulation of p53, a transcription factor shown to be involved in DNA-damage-induced apoptosis, DNA repair, and cell cycle arrest, has been demonstrated to play a role in the development of the advanced stage of the disease [38,39]. In addition, the presence of wild-type (wt) p53 has been demonstrated to have a survival effect on PCa cells after exposure to ionizing radiation, presumably due to the fact that PCa cells exhibit decreased radiation-induced apoptosis [40]. This agrees with what was observed in our study, since the PC3 cell line, which does not possess a functional p53 [32], is the one showing the highest radiosensitivity. These results were also in accordance with the observation that the levels of γ-H2AX foci formed upon irradiation with x-rays decreased with recovery time significantly more in the LNCaP (wt p53) cell line than in the PC3 (p53 null) cell line, reflecting the ability of the cell lines to repair the damage to their DNA [41]. Overall, these observations suggest that ^64^CuCl_2_ might be a good candidate to be used in combination with inhibitors targeting wt p53, in order to increase the radiosensitivity of the PCa cell lines that conserve a functional form of this protein. Interestingly, DNA repair deficiencies have been found to be enriched in advanced metastatic PCa, constituting a potential novel therapeutic target for the disease [42]. In that context, it has been put forth that, in those DNA-repair deficient subset of tumors, the use of drugs that induce lesions unable to be repaired by the mutated systems would be of high therapeutic value [43,44]. Since ^64^CuCl_2_ induces lesions in the DNA that would need to be repaired by the cells, it is plausible that it might also be a therapeutic alternative to treat the tumors that present DNA repair deficiencies. In addition, the observation that non-tumoral cells are able to repair the initial lesions induced to its DNA supports the use of this radiopharmaceutical in a multiple-dose regimen. Such a fractionated treatment has already been used successfully in a preclinical study with an animal model of glioblastoma, which demonstrated that single- and multiple-dose administration was equally effective in terms of reduction of the tumor volume, but the fractionated treatment was the only one that displayed no symptoms of radiotoxicity [19].

In summary, this was the first proof-of-concept cellular study showing that ^64^CuCl_2_ was indeed promising for theranostics of PCa while having the potential to have minimal secondary effects to healthy tissues. Nevertheless, many of the molecular mechanisms underlying the observed behavior need to be further elucidated. Future clinical applications of ^64^CuCl_2_ will also require not only accurate human dosimetric studies, which have started to emerge [31,45], but also accurate micro and nanodosimetric assessments which are sorely lacking.

## 4. Materials and Methods

### 4.1. Cell Culture

Human prostate cancer cell lines (22RV1, DU145, LNCaP, PC3, and VCaP), and the non-tumoral cell line (RWPE-1) were provided by Professor Carmen Jerónimo from the Portuguese Institute of Oncology—Porto, Portugal. 22RV1, DU145, LNCaP, and PC3 cells were routinely grown in RPMI-1640 medium, VCaP cells were grown in DMEM, and RWPE-1 cells were cultivated in KSFM supplemented with 0.05 mg/mL bovine pituitary extract, and 5 ng/mL recombinant human epidermal growth factor. All culture media were further supplemented with 10% heat-inactivated fetal bovine serum, and 1% penicillin and streptomycin (for plate assays only). All culture media and supplements were acquired from Thermo Fisher Scientific (Waltham, MA, USA). Cells were grown at 37 °C in a humidified atmosphere of 5% CO_2_. All cell lines were tested for mycoplasma using the LookOut^®^ mycoplasma PCR Detection Kit (Sigma-Aldrich, Saint Louis, MO, USA), according to the manufacturer’s instructions, and were not used for more than 15 passages at a time after resuscitation.

### 4.2. Production of ^64^CuCl_2_

Copper-64 was produced as a purified, aqueous solution of ^64^CuCl_2_ in 0.1 M HCl, as described in Reference [46]. Prior to the subsequent biological studies, in order to avoid a significant change in the pH of the culture medium upon addition of ^64^CuCl_2_, the pH of the solution was adjusted to ~7 using an appropriate volume of 10 M NaOH and 1 M phosphate buffer (pH 7.2). Owing to the high specific activity of the ^64^CuCl_2_ solution, all the concentrations used in the following biological assays were in the picomolar range.

### 4.3. Cellular and Nuclear Uptake Assays

For cellular uptake assays, cells were seeded at a density of 1.5 × 10^5^ cells/well in 24-well culture plates and allowed to attach overnight. Then, uptake assays were performed as described in Reference [47], using ^64^CuCl_2_ at 0.037 megabecquerel (MBq)/mL. For each assay, cell lysates from four wells were kept at −20 °C and allowed to decay before protein concentration was determined using the DC™ Protein Assay (Biorad, Hercules, CA, USA). The amount of cell-associated radioactivity for each cell line was calculated as the percentage of total activity normalized to the amount of cellular protein. Four technical replicates were used for each time point, and at least three independent uptake experiments were performed. For nuclear uptake assays, cells were seeded at a density of 1.0 × 10^6^ cells/well in 6-well culture plates and allowed to attach overnight. The cells were then incubated with ^64^CuCl_2_ at 0.037 MBq/mL for 3 h before nuclear uptake was determined as previously described in Reference [24]. Two technical replicates were used for each cell line in two independent experiments, and the nuclear uptake was expressed as the percentage of total cellular uptake.

### 4.4. Efflux Assays

Cells were seeded at a density of 1 × 10^5^ cells/well in 24-well culture plates and allowed to attach and grow for 1.5 days. Then, cellular retention was assessed as previously described in Reference [47], after 3 h of incubation with ^64^CuCl_2_ (0.037 MBq/mL) in culture medium. The percentage of cellular retention was calculated using four wells for each time point and two independent experiments.

### 4.5. Protein Extraction

For determination of basal expression of copper transporters, cells grown close to confluence in flasks were lysed in ice-cold CelLytic™ M Cell Lysis reagent (Sigma-Aldrich, Saint Louis, MO, USA), containing a cocktail of protease inhibitors (Roche Applied Science, Penzberg, Germany). The cellular suspension obtained was clarified by centrifugation at 12,000 *g* for 15 min. The resulting supernatant was stored at −20 °C until further use and protein concentration was quantified using the DC™ Protein Assay (Biorad, Hercules, CA, USA). For western blot determination of protein levels after ^64^CuCl_2_ exposure, cells were seeded at a density of 3.0 × 10^5^ cells/well in 6-well culture plates and allowed to attach overnight. Cells were then incubated for 3 h in growth medium containing or not (as a control) 2.8 MBq of ^64^CuCl_2_ and protein extracts were prepared and quantified as described above.

### 4.6. Western Blotting

Forty μg of protein extract were resolved by SDS-PAGE in 4–20% Mini-PROTEAN^®^ TGX™ Precast Protein Gels (Biorad, Hercules, CA, USA) for hCtr1 or 7% acrylamide gels for Atp7A and Atp7B (at 120 V for 1 h 30). After SDS-PAGE resolution, protein extracts were transferred to nitrocellulose membranes for immunoblotting. The membranes were probed with the appropriate primary antibodies, followed by HRP-conjugated secondary antibodies. Western blot signal was detected by enhanced chemiluminescence using Pierce™ ECL Western Blotting Substrate (Thermo Fisher Scientific, Waltham, MA, USA). Primary antibodies used in this study were: 1:2500 rabbit anti-Atp7B (Abcam), 1:6500 chicken anti- Atp7A (Abcam), 2 μg/mL rabbit anti-hCTR1 (Aviva Systems Biology), and 1:25000 mouse anti-actin (Sigma-Aldrich), used as a loading control. All secondary antibodies (anti-rabbit (Abcam, Cambridge, UK), anti-chicken (Abcam, Cambridge, UK), and anti-mouse (Biorad, Hercules, CA, USA)) were used at a 1:3000 dilution.

### 4.7. γ-H2AX Assay and Foci Analysis

22RV1, LNCaP, PC3, and RWPE-1 cells were seeded at a density of 2.0 × 10^4^, 8.5 × 10^4^, 1.5 × 10^4^, and 3.0 × 10^4^ cells/well, respectively, in an 8-well chamber slide (Merck Millipore, Burlington, MA, United States) and allowed to attach overnight. Cells were incubated or not (as a control) with 1.1 or 2.8 MBq of ^64^CuCl_2_ in 500 μL of medium for 3 h at 37 °C. γ-H2AX immunostaining and visualization was performed as previously described in Reference [24]. Image analysis of γ-H2AX foci was performed in ImageJ with a custom macro. A minimum of 50, but usually ≥ 100) nuclei were analyzed per experiment, in triplicates.

### 4.8. Cytokinesis-Blocked Micronucleus (CBMN) Assay

PC3 and RWPE-1 cells were seeded at a density of 1.5 × 10^5^ and 2.0 × 10^5^ cells/well, respectively, in 6-well culture plates containing coverslips, and allowed to attach and grow for 24 h. Cells were then incubated for 3 h in a growth medium containing or not 2.8 MBq of ^64^CuCl_2_, before ^64^CuCl_2_ was removed, and fresh medium was added. Cytochalasin B (1 μg/mL) was added 17 h after exposure to ^64^CuCl_2_, and the cells were incubated for an additional 28 h. Then, cells were fixed and stained, and the slides were visualized as previously described in Reference [48]. For each experiment, 1000 binucleated cells (BN) were scored and two independent experiments were performed for each cell line.

### 4.9. Cell Proliferation and Colony Formation Assay

PC3 and RWPE-1 cells were seeded in 6-well culture plates (50, 100, and 200 cells for control conditions, and 200, 400, and 1000 cells per well for ^64^CuCl_2_ exposure) and allowed to attach for 6 h. For the ^64^CuCl_2_ exposure plate, a volume corresponding to 2.8 MBq in 1.5 mL of medium was added for 3 h. The cells were left to grow until colonies of at least 50 cells had formed in control wells (10–14 days). Then, all plates were fixed and colonies stained as described above for the MN assay. Only colonies with more than 50 cells were counted, and three independent experiments were performed for each cell line.

### 4.10. Statistical Analysis

All data were shown as mean values ± standard error of the mean (S.E.M.). Statistical analysis was carried out using GraphPad Prism 6 software. Statistical differences between treatment and control samples were assessed using two-tailed Student’s t-test. Differences among more than two groups were assessed by one-way ANOVA followed by Tukey’s test when appropriate. The threshold for statistical significance was set to *p* = 0.05.

## Supplementary Materials

The following are available online at http://www.mdpi.com/1420-3049/23/11/2944/s1, Figure S1: Expression of hCtr1 in human prostate cell lines. (A) Western analysis was used to examine the basal protein expression levels of the copper importer hCtr1 in the panel of PCa cell lines under study, which identified two distinct bands (of roughly 35 kDa and 70 kDa). Actin was used as a loading control. Statistical significance was calculated using one-way ANOVA, followed by Tukey’s test (* *p* ≤ 0.05, ** *p* ≤ 0.01). The results were calculated from independent biological replicates (n ≥ 4) and are given as the mean ± S.E.M.

## Abbreviations

ATSM	diacetyl-bis N4-methylthiosemicarbazone
BN	binucleated
CT	computed tomography
CBMN	cytokinesis-block micronucleus
DSBs	double-strand breaks
LET	linear energy transfer
MBq	megabecquerel
MN	micronucleus
MNi	micronuclei
PBS	phosphate-buffered saline
PCa	prostate cancer
PET	positron emission tomography
PET/CT	positron emission tomography/computed tomography
