# Supplementary material for: Radiobiological Characterization of 64CuCl2 as a Simple Tool for Prostate Cancer Theranostics
Source: Molecules. 2018 Nov 11;23(11):2944. doi: 10.3390/molecules23112944 (PMC6278521; doi:10.3390/molecules23112944)
Supplement: Supplementary file 1 [file molecules-23-02944-s001.pdf]

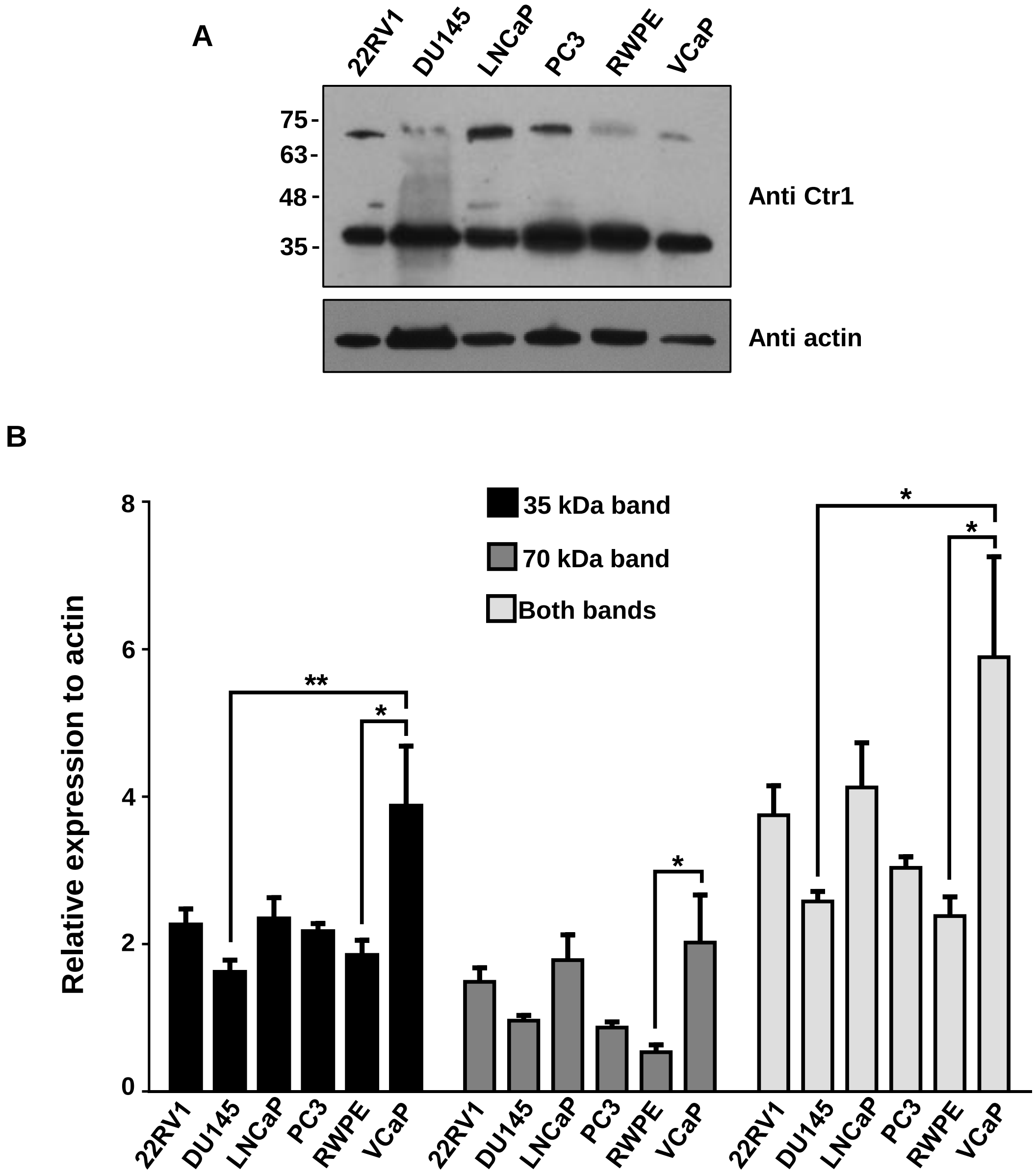

Figure S1: Expression of hCtr1 in human prostate cell lines. (A) Western analysis was used to examine the basal protein expression levels of the copper importer hCtr1 in the panel of PCa cell lines under study, which identified two distinct bands (of roughly 35 kDa and 70 kDa). Actin was used as a loading control. Statistical significance was calculated using one-way ANOVA, followed by Tukey's test (* $p \leq 0.05$, ** $p \leq 0.01$). The results were calculated from independent biological replicates ($n \geq 4$) and are given as the mean ± S.E.M.
